# Understanding Surface Interaction and Inclusion Complexes between Piroxicam and Native or Crosslinked β-Cyclodextrins: The Role of Drug Concentration †

**DOI:** 10.3390/molecules25122848

**Published:** 2020-06-19

**Authors:** Giuseppina Raffaini, Fabio Ganazzoli

**Affiliations:** 1 Department of Chemistry, Materials, and Chemical Engineering “Giulio Natta”, Politecnico di Milano, Piazza L. Da Vinci 32, 20131 Milano, Italy; fabio.ganazzoli@polimi.it; 2 INSTM, National Consortium of Materials Science and Technology, Local Unit Politecnico di Milano, 20133 Milano, Italy

**Keywords:** β-cyclodextrin, inclusion complexes, solubilization, nanosponge, nanocarriers, pharmaceutical applications, molecular recognition phenomena, molecular dynamics simulations, drug delivery, drug concentration

## Abstract

Drug concentration plays an important role in the interaction with drug carriers affecting the kinetics of release process and toxicology effects. Cyclodextrins (CDs) can solubilize hydrophobic drugs in water enhancing their bioavailability. In this theoretical study based on molecular mechanics and molecular dynamics methods, the interactions between β-cyclodextrin and piroxicam, an important nonsteroidal anti-inflammatory drug, were investigated. At first, both host–guest complexes with native β-CD in the 1:1 and in 2:1 stoichiometry were considered without assuming any initial a priori inclusion: the resulting inclusion complexes were in good agreement with literature NMR data. The interaction between piroxicam and a β-CD nanosponge (NS) was then modeled at different concentrations. Two inclusion mechanisms were found. Moreover, piroxicam can interact with the external NS surface or with its crosslinkers, also forming one nanopore. At larger concentration, a nucleation process of drug aggregation induced by the first layer of adsorbed piroxicam molecules is observed. The flexibility of crosslinked β-CDs, which may be swollen or quite compact, changing the surface area accessible to drug molecules, and the dimension of the aggregate nucleated on the NS surface are important factors possibly affecting the kinetics of release, which shall be theoretically studied in more detail at specific concentrations.

## 1. Introduction

Cyclodextrins (CDs) are cyclic oligosaccharides known for more than 100 years, recognized as pharmaceutical adjuvants for the past 20 years [1,2,3]. Thanks to their capability to form noncovalent water-soluble complexes, they are useful as functional excipients for solubilization, delivery, and greater bioavailability of drugs in many different applications [4,5]. Cyclodextrins have an approximatively truncated cone shape and may be described as a bucket with a hydrophilic outer surface and a hydrophobic central cavity. CDs may thus form noncovalent host–guest complexes hosting hydrophobic drugs and some hydrophilic guests. The intermolecular interactions are due to electrostatic interactions, weak van der Waals contributions, hydrogen bonds with secondary and/or primary rims, and hydrophobic interactions within the cavity and the nonpolar groups or π electrons of the guest. In an aqueous solution, natural CDs can interact. The more water-soluble cyclodextrin derivatives have a low tendency to form aggregates [6]. Cyclodextrin derivatives can be hydrophilic or relatively lipophilic based on their substitution and these properties can give insights into their ability to act as permeability enhancers. Natural CDs are α-CD, β-CD, and γ-CD composed of six, seven, and eight units of d-glucopyranose, respectively, with α-1,4 linkages. Low cost, easy synthetic accessibility, and suitable cavity size (0.60−0.65 nm) for the inclusion of small- and medium-sized drugs result in the wide use of β-cyclodextrin (β-CD) in pharmaceutical and food industries, on the early stages of pharmaceutical applications. Drugs often have various problems: they are hydrophobic, with low solubility in water and low stability either in vivo or in vitro. All these factors reduce the therapeutic effect of the drug. In order to overcome these problems, β-CD has been widely used. The drug can interact with the β-CD hydrophobic cavity and form host–guest inclusion complexes, possibly with different stoichiometries. Therefore, CDs enhance the bioavailability of insoluble drugs by increasing their solubility, dissolution, drug permeability, by making the drug available at the surface of biological barriers so that the in vivo and in vitro stability increases.

Over the past 20 years, it has been shown that CDs and CD complexes self-associate to form an aggregate or micelle-like structures [7,8]; sometimes, it is very difficult to detect them. Furthermore, the formation of drug/CD complex nanoparticles appears to increase the ability of CDs to enhance drug delivery through some mucosal membranes. Recently, chemically modified β-CD derivatives have been synthesized [9,10,11,12,13,14] and also theoretically studied [15] in order to improve cyclodextrin interactions with hydrophobic drugs and to enhance drug release through cell membranes. One of the possible modifications is to chemically bind aliphatic chains of different lengths on the primary or secondary CD rim in order to obtain amphiphilic cyclodextrins (aCD) [9]. This modification allows increasing the cyclodextrin interactions with biological membranes, improving their interaction with hydrophobic drugs, and inducing a higher self-assembly capacity in aqueous solutions compared to native β-CD [16]. This last property has been used to obtain β-CD-based nanosized carriers. Again, in the last years, new CD derivatives enhancing solubility and bioavailability have been synthetized considering both linear polymerized β-CDs [10] or crosslinked systems, such as β-CD nanosponges (β-CD NS) and chemically modified β-cyclodextrin derivatives [11,12,13,14].

**Scheme 1:**
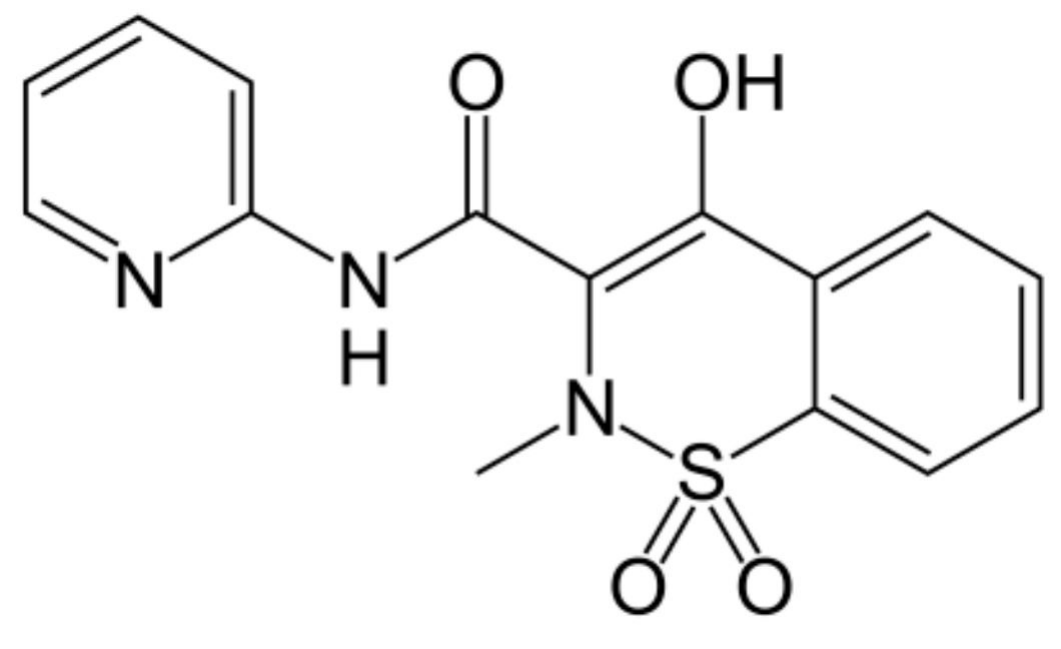
Chemical structure of the piroxicam drug molecule.

Piroxicam (PX) is one of the most efficient nonsteroidal anti-inflammatory agents widely used for the treatment of rheumatoid arthritis, osteoarthritis, and acute pain in musculoskeletal disorders (see Scheme 1).

Because of its very low solubility in gastrointestinal fluids, it has poor bioavailability after oral administration. The formation of inclusion complexes with β-CD may be a useful strategy in order to overcome solubility and bioavailability problems that are very important, in particular, for pediatric and geriatric patients. In 1992, Fronza et al. [17] reported an NMR study of a 1:1 β-CD/PX inclusion complex. Significant nuclear Overhauser effects were observed between inner protons of CD and the protons of both aromatic rings of the piroxicam molecule. The data indicated the possibility of having two different inclusion complexes with two different equilibrium constants within the investigated range of concentrations; furthermore, at smaller concentrations, the complex was found to be completely dissociated. Therefore, the possibility of identifying two types of host–guest inclusion compounds and the influence of the concentration for the stability and formation process are important factors that should be better investigated. In 2003, Guo et al. [18] investigated β-CDs and PX host–guest complexes using steady state fluorescence and NMR techniques, again indicating a strong interaction between the hydrophobic drug and the hydrophobic cavity of β-CDs, in particular forming stable complexes in a 1:1 stoichiometry and probably in a 1:2 stoichiometry. In order to enhance both the solubility and the fast release of PX, in 2019, Dharmasthala et al. [19] proposed a very interesting formulation for an oral film containing the β-CD/PX inclusion complex, studying the dissolution process in vitro, indicating fast drug diffusion, and permitting to obtain a better therapeutic efficiency.

Over the past 20 years, molecular mechanics (MM) and molecular dynamics (MD) simulations have been demonstrated to be a useful tool in order to atomistically investigate and describe non-covalent interactions in different systems ranging from protein adsorption on surfaces of biomaterials to formation and self-aggregation of host–guest complexes, including their stoichiometry and self-aggregation in an nonpolar solvent or in water [20,21,22,23,24,25,26]. Molecular simulation studies offer great insights into these phenomena in very good agreement with experimental data [20,27,28,29].

In this paper, a theoretical study of the host–guest inclusion complexes between β-CD and PX is reported with no a priori assumption about the inclusion stoichiometry and geometry in a 1:1 and in a 2:1 stoichiometry as suggested by NMR data. Then, the interaction with a nanosponge (NS) model containing β-CDs connected with a pyromellitic dianhydride (PMA) crosslinker [30] is studied (PMA NS). The NS was generated by linking 8 β-CDs (Model 2 in [29]) through PMA moieties (see Scheme 1 in [30]). Accordingly, each CD carries two PMA linking agents bound to primary hydroxyls at diametrically opposite sides of the macrocycle. Increasing the number of drug host molecules, a ratio between the number of β-CDs in the NS model and the number of PX molecules (8:4, 8:8, 8:16, 8:40), i.e., a β-CD/PX ratio of 2:1, 1:1, 1:2, 1:5 was investigated. The results for the interaction with the NS at different drug concentrations are then reported. The conformational changes during the MD runs are also investigated. The types of inclusion complexes also formed in a crosslinked system and the surface interaction will be highlighted. The drug concentration influencing formation of the host–guest complexes and the surface β-CD/drug interactions related to the NS flexibility as well as the drug self-association will also be described.

## 2. Results and Discussion

### 2.1. β-CD/PX Interaction

In this section, the β-CD/PX interaction forming host–guest inclusion complexes in 1:1 and 2:1 stoichiometries in implicit water is studied. The theoretical results are reported and discussed in comparison with NMR experiments in the literature.

#### 2.1.1. β-CD/PX Interaction: Complex Formation in a 1:1 Host–Guest Stoichiometry

**Figure 1:**
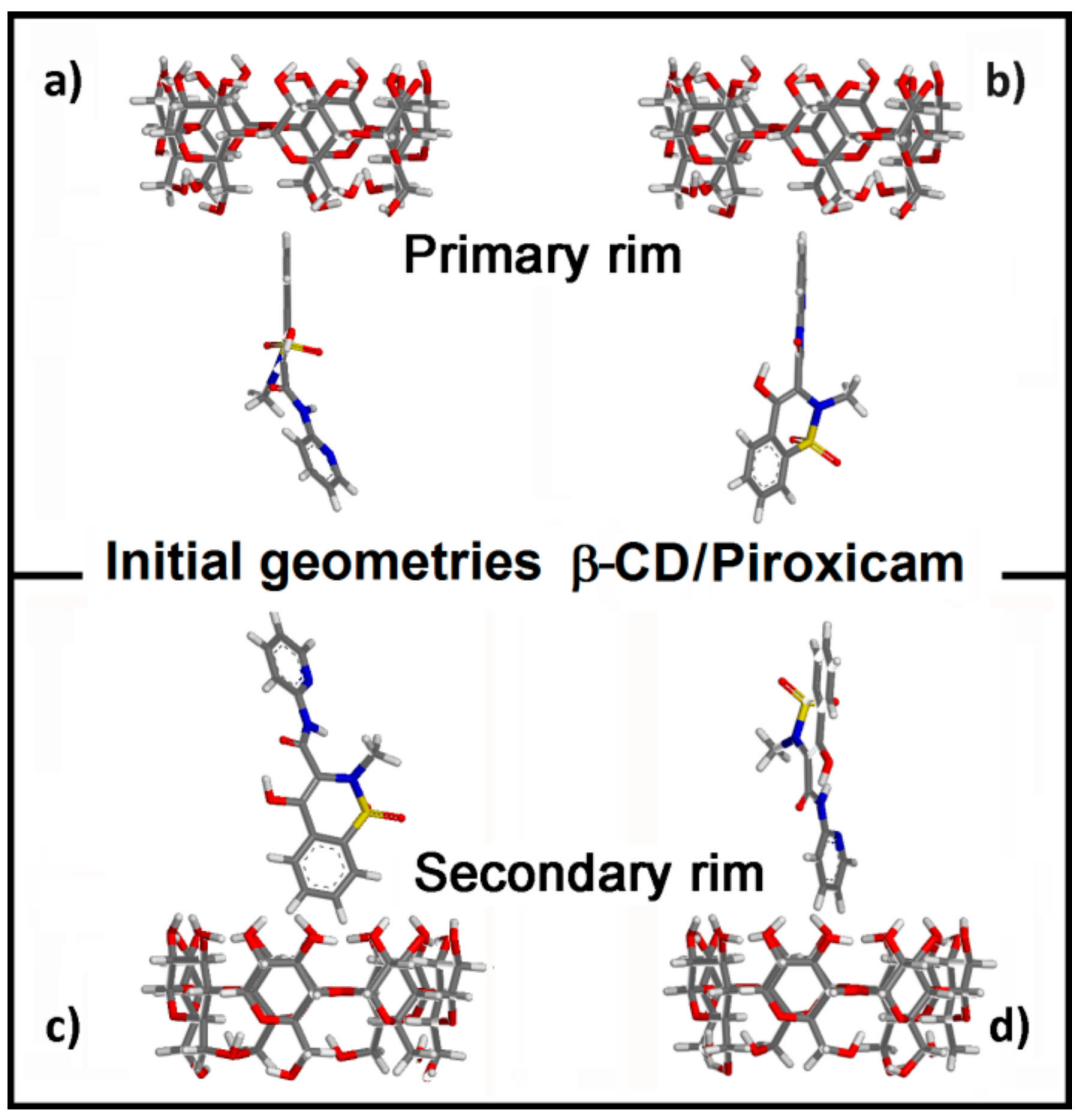
Side view of the four initial non-optimized geometries studied for the interaction between PX and β-CD with the primary rim and with the secondary rim at the top and the bottom, indicated respectively as (**a**–**d**). The two molecules are colored by atoms. Color codes: C atoms are grey, O atoms are red, N atoms are blue, S atoms are yellow, and H atoms are white.

At first, the PX conformation was studied, and then the β-CD reported by Raffaini [22] was considered for the interaction between piroxicam and β-CD in a 1:1 stoichiometry considering possible host–guest inclusion arrangements without assuming any a priori inclusion complexes. Using a simulation protocol adopted in the previous work [20,21], four different starting geometries between piroxicam and the primary and secondary rim of β-CD are considered in the 1:1 stoichiometry as reported in Figure 1.

**Figure 2:**
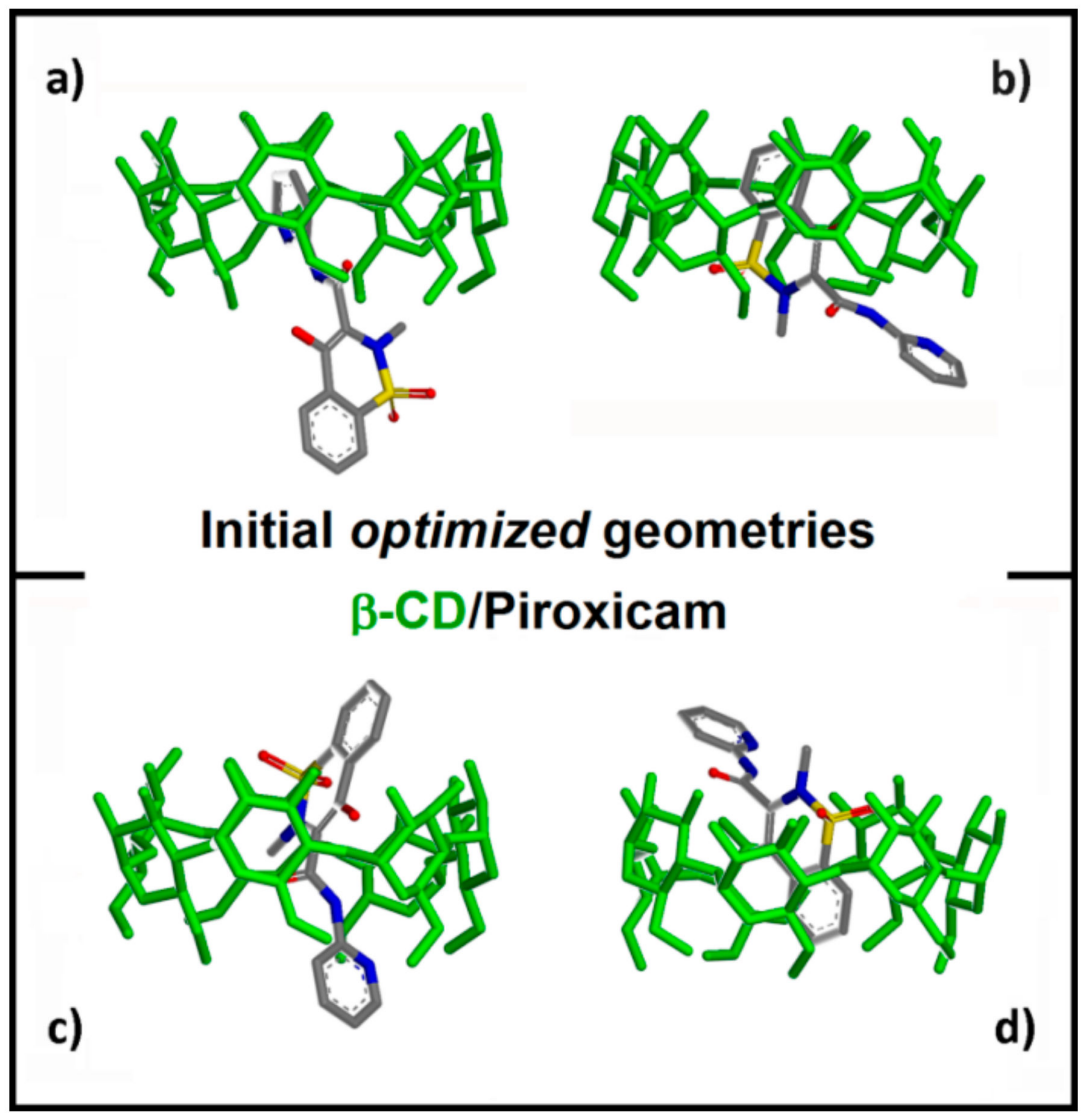
Side view of the 1:1 host–guest complexes formed by PX with β-CD, in the initial optimized geometries found after the MM calculations, starting from the four geometries reported in Figure 1 indicated respectively as (**a**–**d**). The β-CDs are green, the PX molecule is colored by atoms. Color codes: C atoms are grey, O atoms are red, N atoms are blue, S atoms are yellow, and H atoms are omitted for clarity.

These four initial geometries were optimized and after the initial energy minimizations, favorable host–guest complexes were formed. The final optimized arrangements found after these MM calculations are shown in Figure 2.

**Figure 3:**
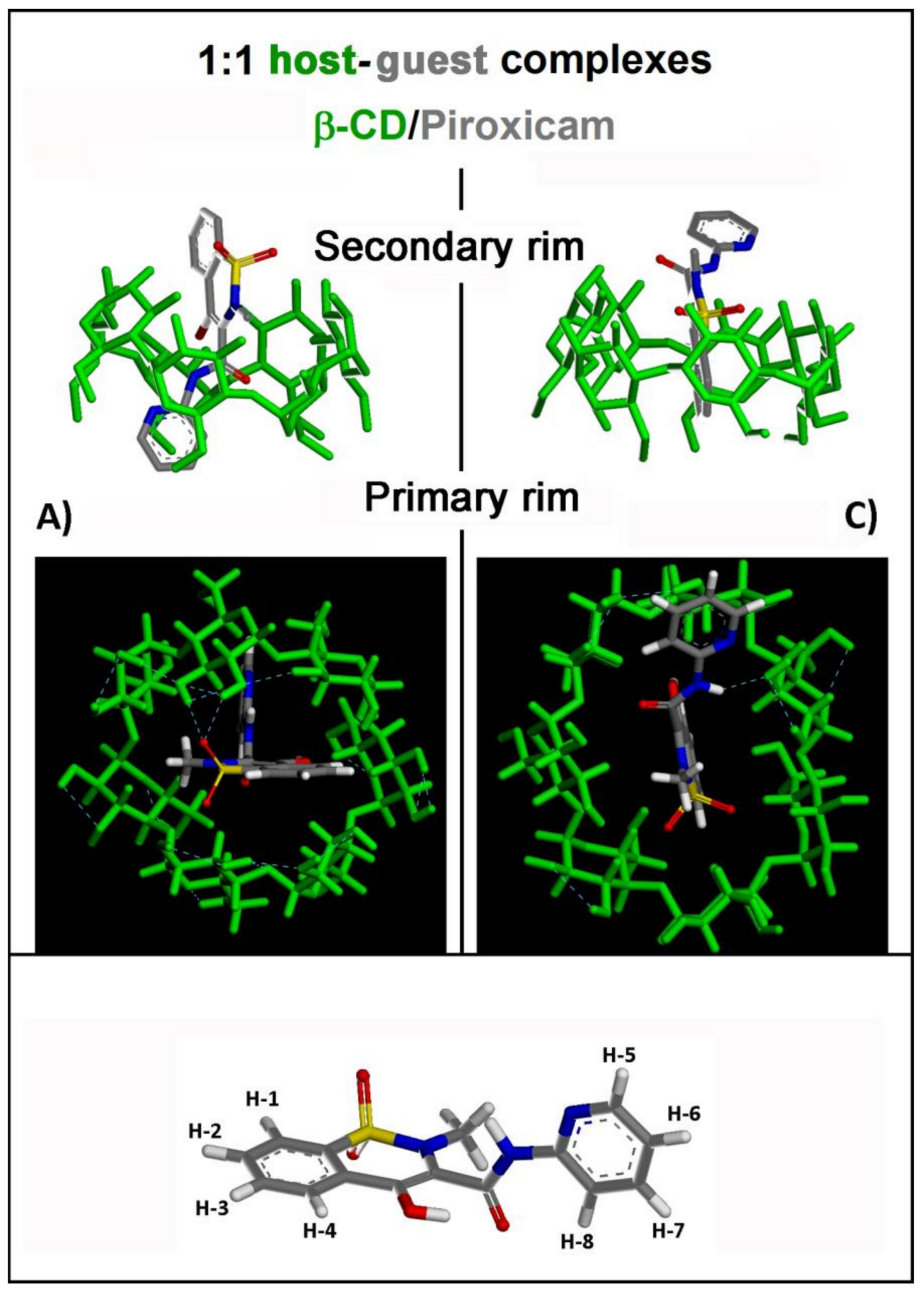
The side view and the top view with intermolecular H-bonds (light blue dotted line) of the 1:1 host–guest complexes formed by PX with β-CD (**A**) in the most stable and (**C**) in the less stable optimized geometries found after the MM, MD calculations. The β-CD is green, the PX molecule is colored by atoms (color codes: C atoms are grey, O atoms are red, N atoms are blue, S atoms are yellow, and in the side view, H atoms are omitted for clarity). Below, the conformation of the optimized isolated PX is reported with the diagnostic hydrogen highlighted.

After these initial energy minimizations, starting from all the four different geometries, MD runs were performed in implicit water and the final conformations assumed by the system at the end of each MD run were analyzed. Two different inclusion complexes were found, the most and the less stable host–guest inclusion complexes in the 1:1 stoichiometry being reported in Figure 3, while Table 1 reports the potential energy, the calculated interaction energies, and the information about the intermolecular and β-CD intramolecular H-bonds for all four optimized geometries. In Figure S1, the two metastable geometries reported in Table 1 indicated as B and C D are shown. The animation of the MD runs is shown in Supplemtary Information SI.

As reported by Fronza et al. [17] using NMR experiments, two different inclusion complexes having different stability were found. The most stable geometry reported in Figure 3 (geometry A) displays the pyridyl aromatic part of PX included in the hydrophobic cavity of β-CD near the primary rim interacting with its hydrogens, while the second geometry of Figure 3 (geometry C) is less stable, and displays the pyridyl moiety included in the hydrophobic β-CD cavity facing the secondary rim interacting with the latter hydrogens. The most stable geometry has an interaction energy equal to −173.2 kJ/mol, while the other one is less stable by 10 kJ/mol; similar favorable interactions stabilize these host–guest complexes thanks to the hydrophobic interaction in the β-CD cavity and to the H-bonds at the primary and especially at the secondary rim. It is important to underline that these theoretical results are in good agreement with the NMR data reported by Fronza et al. [16] who proposed two different inclusion compounds. It is interesting to note that these two inclusion complexes are also found when β-CD are crosslinked in the PMA NS model as it will be reported later in Section 2.2. Guo et al. [18] proposed a partial inclusion due to the absence of a Nuclear Overhauser Effect NOE of H-2, H-3 guest hydrogens (see Figure 3, H-2 and H-3 are the hydrogens of the PX phenyl ring) and diagnostic hydrogen β-CD, suggesting that the dimension of β-CD is too small to host the whole PX. In fact, in the most stable geometry with a 1:1 stoichiometry reported in Figure 3 (geometry A), the hydrogens of the phenyl ring are far from the β-CD cavity; only in the less stable and less populated inclusion complex, the phenyl ring is in the midst of the hydrophobic cavity, relatively closer to H-5’ protons, while the pyridyl moiety is exposed to the solvent far from the secondary rim (geometry C in Figure 3). Guo et al. reported the 2D-ROESY (Rotating-Frame Overhauser Effect Spectroscopy) spectrum. In this spectrum, strong sizable contacts between the guest H-6, H-7, and H-8 protons (the hydrogens of the pyridyl moiety) and the host H-3’ and H-5’ are shown, probably due to deeper insertion, indicating that both phenyl and pyridyl rings are in the β-CD cavity. For this reason, Guo also suggested the presence of a β-CD and PX complex in a 2:1 stoichiometry with CDs facing their secondary rims. It will be important to carry out new reliable NMR experiments for the determination of the structure of cyclodextrin inclusion complexes in a solution [31], in particular, off-resonance ROESY or T-ROESY (Transverse Rotating-frame Overhauser Enhancement Spectroscopy) experiments must be carried out in order to determine the structure of complexes in a solution. In this work, increasing the CD concentration, a theoretical study of host–guest complexes in a 2:1 stoichiometry was carried out as reported in the next Section.

#### 2.1.2. β-CD/PX Interaction: Complex Formation in a 2:1 Host–Guest Stoichiometry

**Figure 4:**
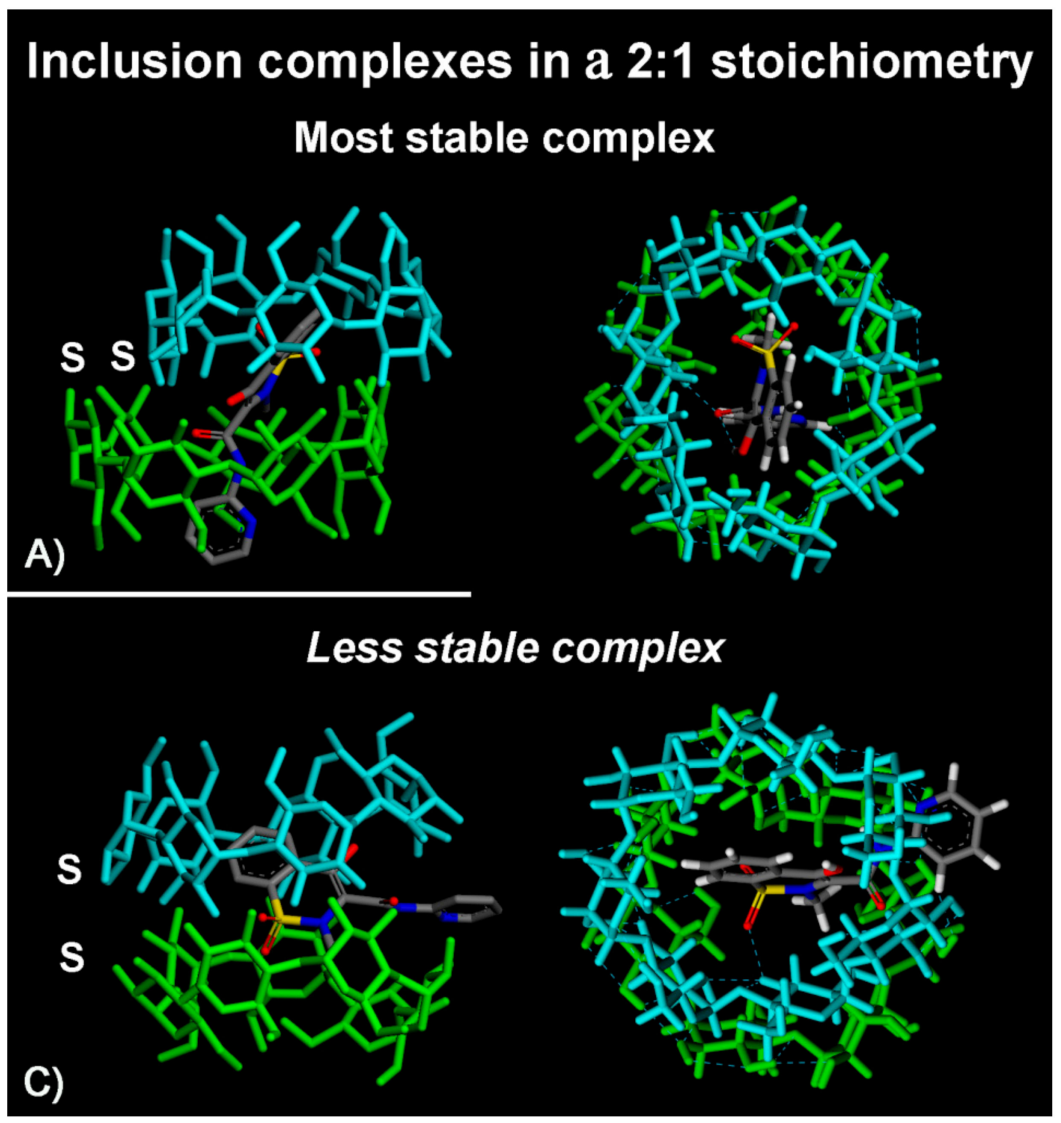
The side view (left) and the top view with H-bonds (light blue dotted lines) (right) of the 2:1 host–guest complexes formed by PX with two β-CDs, (**A**) in the most stable and (**C**) in the less stable optimized geometries found after the MD runs. The β-CD of the first 1:1 complex is in green, the second one facing the secondary rim is in light blue. The piroxicam molecule is colored by atoms. Color codes: C atoms are grey, O atoms are red, N atoms are blue, S atoms are yellow. In the side view, the H atoms are omitted for clarity.

The β-CD/PX in a 2:1 host–guest geometry was investigated considering both the most and the less stable inclusion complexes in a 1:1 stoichiometry reported in Figure 3: four different interaction geometries between these complexes and another β-CD facing both the secondary and the primary rim were considered (Figure S2). After the MD runs and final energy minimizations, the optimized geometries for the most stable and the less stable arrangements are shown in Figure 4. In Figure S3, the two metastable geometries are reported.

In both cases, the dimers formed facing the secondary rims of two CDs display a more favorable interaction energy than the 1:1 inclusion complex (see Table 2), indicating a stabilization more than twice as large. Only few intermolecular β-CD/drug H-bonds were found in these dimers, one in the most stable geometry and two in the less stable one involving not only PX oxygen atoms, but also the nitrogen of the pyridyl moiety. As proposed by Guo et al. from the 2D-ROESY spectrum, PX can effectively interact with two CDs facing their secondary rims in the most stable complexes in a 2:1 stoichiometry. The concentration of β-CD in a solution as indicated by Fronza et al. and by Guo et al. influences the type of the inclusion complex, so that it becomes important to study the variable concentration of host molecules [6,7,8]. It will be necessary to confirm the presence of the 2:1 complex or others complexes by Isothermal Titration Calorimetry (ITC) experiments [32].

### 2.2. PMA β-CD NS Model/PX Interaction at Different Concentrations

In this Section, the PX interaction with a nanosponge (NS) model containing β-CDs connected by a pyromellitic dianhydride PMA crosslinker [30] is investigated. The NS model was generated by linking 8 β-CDs (Model 2 in [30]) through PMA moieties (see Scheme 1 in [30]). Accordingly, each CD carries two PMA linking agents bound to a primary hydroxyl at diametrically opposite sides of the macrocycle. An increasing number of host molecules was considered, with β-CD:PX equal to 8:4, 8:8, 8:16, 8:40, therefore, with β-CD:PX in a 2:1, 1:1, 1:2, 1:5 ratio.

**Figure 5:**
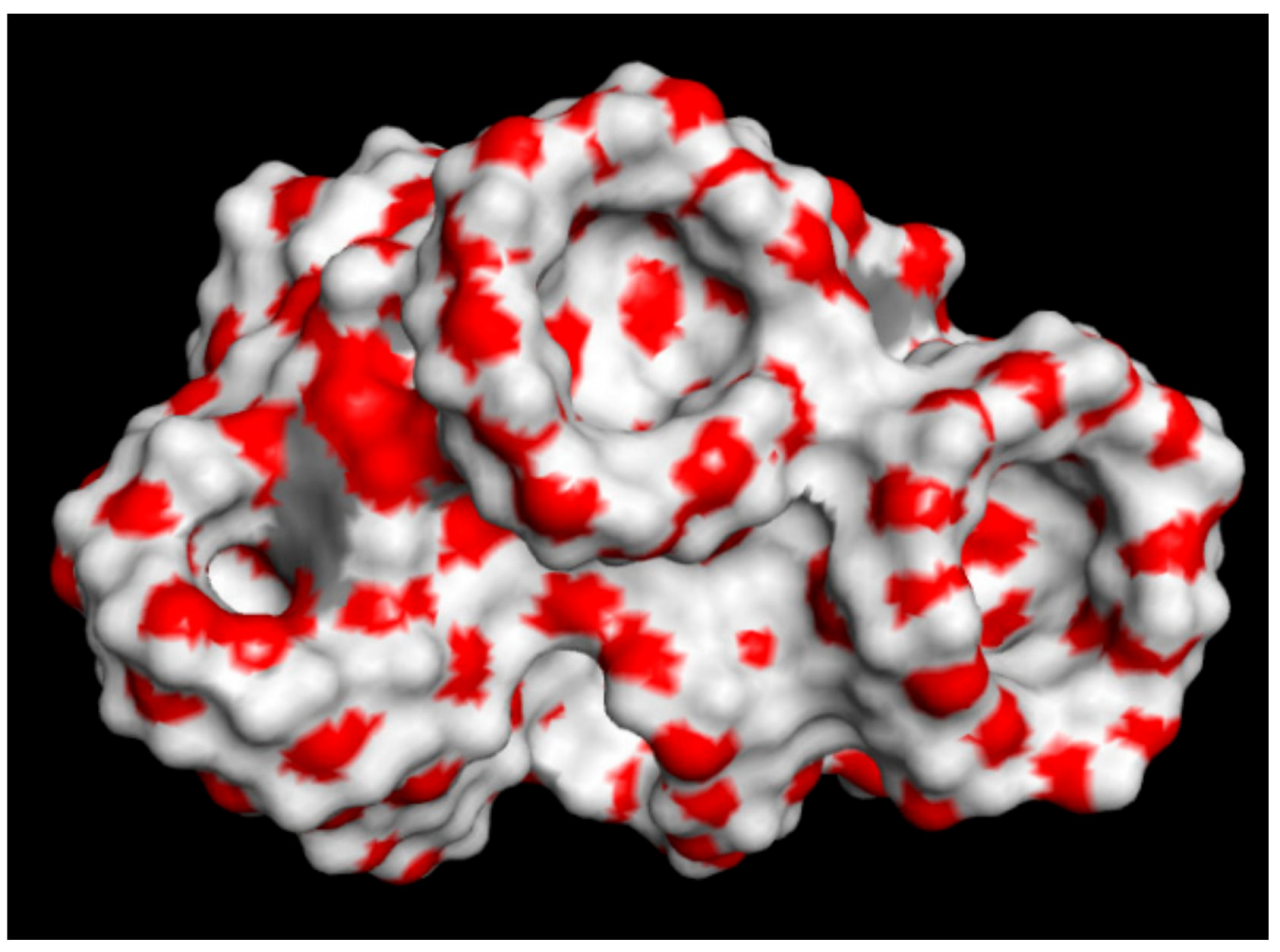
The solvent-accessible surface area of a β-CD NS (β-cyclodextrin Nanosponge) model with a pyromellitic dianhydride (PMA) crosslinker as reported in Model 2 in [29] colored by atoms. Color codes: C atoms are grey, oxygen atoms re red, and hydrogen atoms are white.

Since it will be relevant in the following, it is important to report that the solvent-accessible surface area calculated for the β-CD NS model reported in Figure 5 is equal to 4,315 Å^2^ and the radius of gyration is equal to 12.5 Å. In the following, the interaction between the PMA β-CD NS model [30] and PX molecules considered at smaller and larger concentrations were studied and the results are reported and discussed.

#### 2.2.1. NS/PX Interaction: 8 β-CDs in the NS Model and 4 PX Molecules (β-CD/Drug in a 2:1 Stoichiometry)

**Figure 6:**
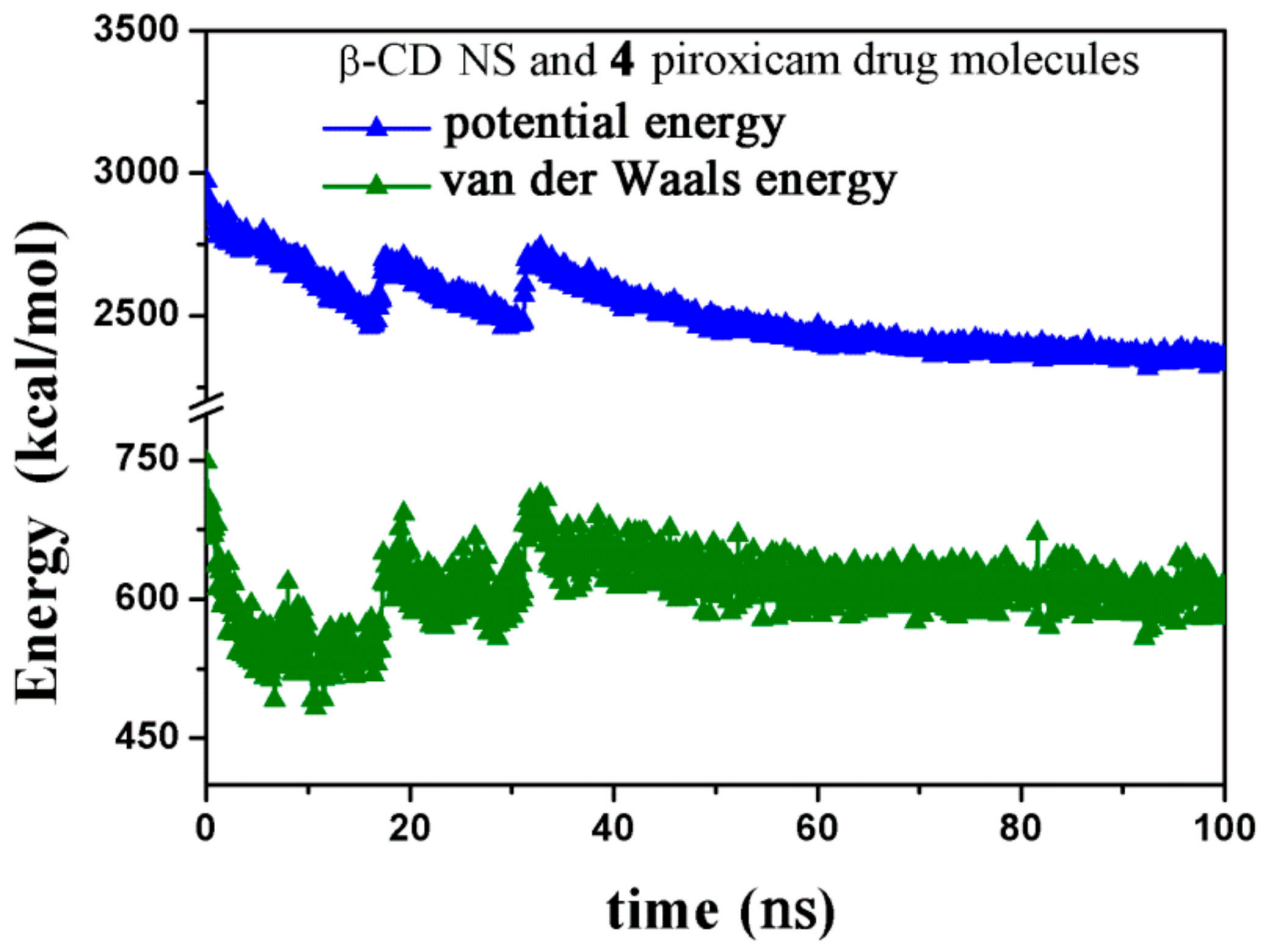
The potential energy and the van der Waals contribution calculated during the MD run for the NS model interacting with 4 piroxicam molecules.

**Figure 7:**
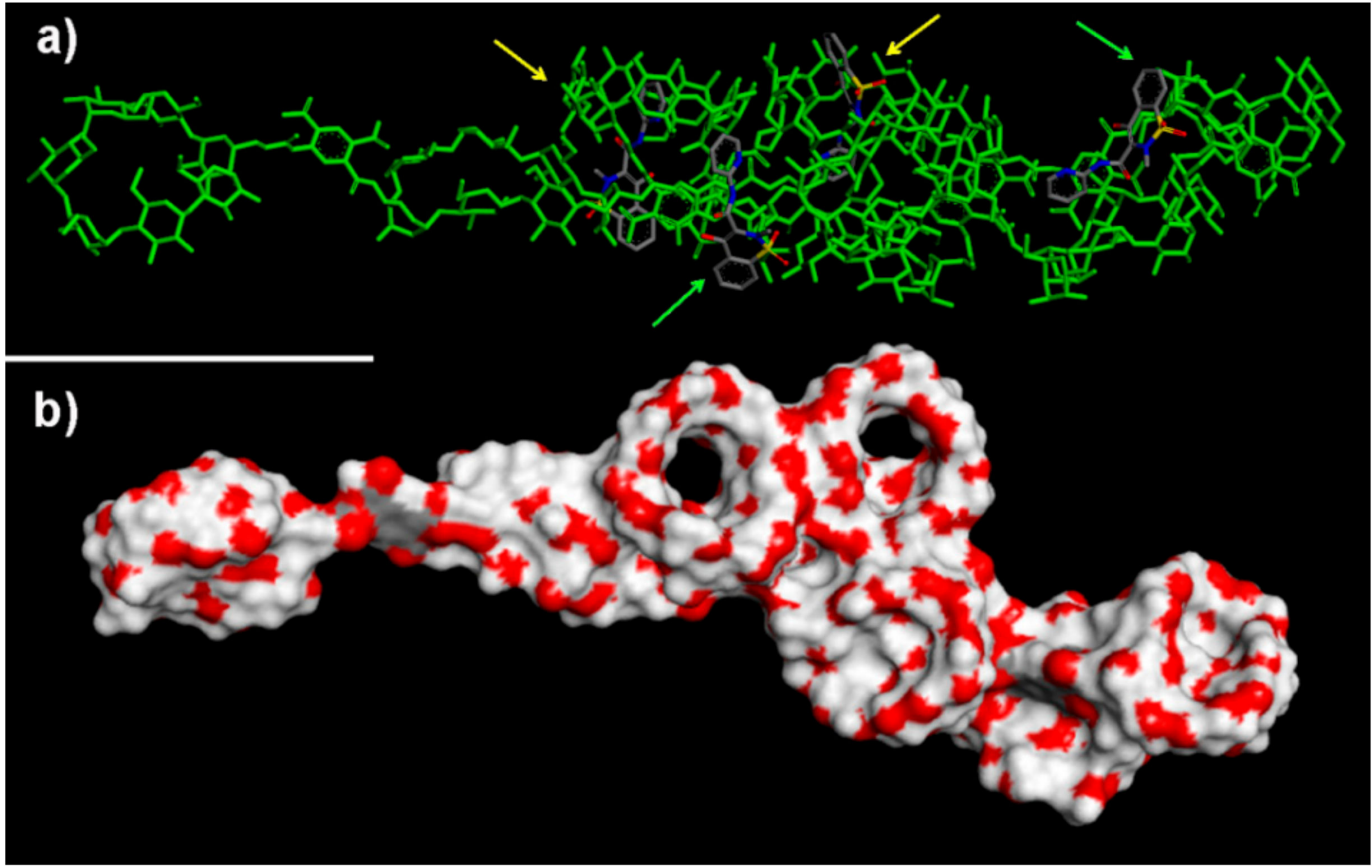
Panel (**a**) shows the optimized geometry assumed by the NS/4 PX molecules system at the end of the MD run displaying two included molecules in a 1:1 stoichiometry (see the yellow arrows) and two PX molecules interacting on the external surface of the NS model. The NS is in green for clarity and the PX molecules are colored by atoms, see the color codes in Figure 3. Panel (**b**) shows the solvent-accessible surface area without the PX molecules for clarity (see the color codes in Figure 5).

Using the simulation protocol proposed in the previous work [20,21,22], as the first step, energy minimization of the NS model and 4 PX molecules, considering the β-CD/drug in a 2:1 stoichiometry, was carried out starting from a random disposition. An MD simulation run was then performed in order to understand possible conformational changes and interactions, in particular, the possible inclusion complexes or the surface interaction with the exposed PMA β-CD NS atoms. In Figure 6, the potential energy and the van der Waals contribution calculated during the MD run are reported. After an initial energy decrease, some conformational changes of the nanosponge take place in order to maximize its interactions with the four PX molecules, increasing the surface area accessible to the drug molecules. It should be noted that hydrophobic drug/drug interactions are absent. The final optimized conformation assumed by the system is reported in Figure 7. See the animation file of the MD run in SI.

It is interesting to note that upon removal of the PX molecules as shown in panel b) of Figure 7, the solvent-accessible surface area of the NS in the optimized final geometry is equal to 5,263 Å^2^, with an increase of about 20% compared to the starting geometry, while the radius of gyration increases by more than 90% to a value of 23.3 Å. It is well-known that β-CD nanosponges are able to swell in a water solution [14,30]. They are very flexible systems that can have significant conformational changes in order to maximize the favorable interactions with drugs. Significant flexibility was displayed by this β-CD crosslinked system during the MD run. The final optimized geometry reported in panel a) of Figure 7 displays an evident elongation of the NS structure interacting with PX molecules forming two different host–guest complexes (see the yellow arrows) in a 1:1 stoichiometry studied and reported in Section 2.1; one complex displays the hydrogens of the pyridyl part preferentially in the β-CD cavity and the phenyl part far from the hydrophobic cavity and the second one displays the pyridyl part far from the β-CD cavity. Interestingly, these geometries are very similar to the two inclusion complexes considering the native β-CD discussed in Section 2.1.1 and reported in Figure 3 (see geometries A and C in Figure 3). Again, it is important to note that the other two piroxicam molecules interact with the external part of the NS, in particular, with the exposed crosslinker (see the green arrows in Figure 7).

#### 2.2.2. NS/PX Interaction: 8 β-CDs in the NS Model and 8 PX Molecules (β-CD/Drug in a 1:1 Stoichiometry)

**Figure 8:**
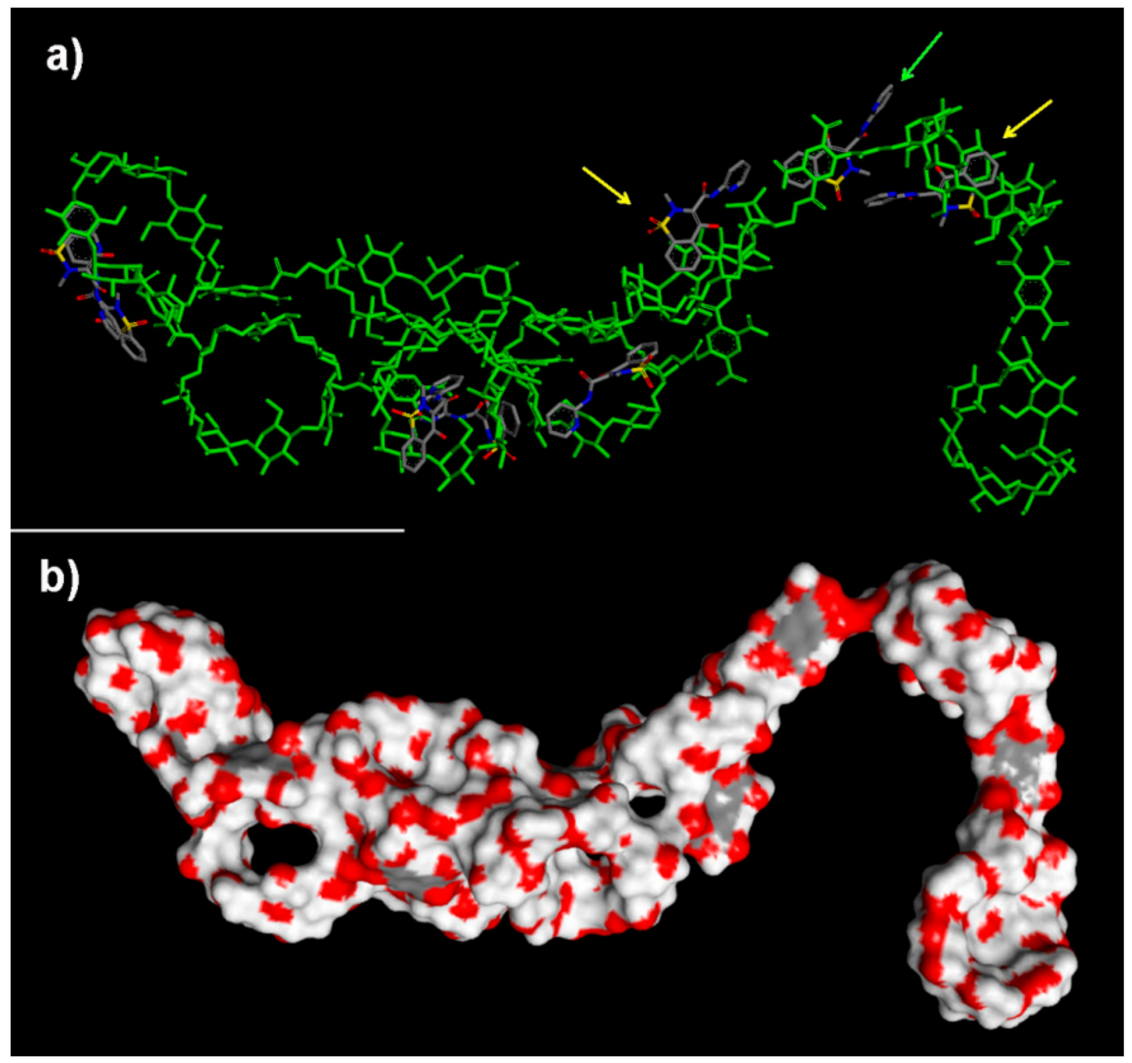
In the Figure, panel (**a**) shows the optimized geometry assumed by the NS/8 PX drug molecules system at the end of the MD run displaying two types of interactions with hydrophobic cavities (see the yellow arrows) and six PX molecules interacting on the external surface of the NS. The NS is in green for clarity and the PX molecules are colored by atoms according to the color codes in Figure 3. Panel (**b**) shows the solvent-accessible surface area without the PX molecules for clarity (see the color codes in Figure 5).

In this Section, the interaction between PMA β-CD NS with PX was studied considering 8 drug molecules. The NS contains 8 β-CD [30], so that the β-CD/drug in a 1:1 stoichiometry is investigated. Using the same strategy as before, after the MD run in the final optimized geometry reported in Figure 8, PX was found to interact both with the NS in the accessible hydrophobic cavities and with the NS surface. The NS shows large conformational changes in order to adsorb the PX molecules well. Interestingly, at this concentration, possible drug/drug hydrophobic interactions take place. In the final optimized geometry, again, two types of inclusion complexes are found together with the surface interaction on the exposed atoms of this flexible crosslinked system. As already found for the smaller concentration, the nanosponge maximizes the possible interaction with a drug.

The NS becomes well-elongated due to the favorable interactions with the PX drug forming two inclusion complexes (see the yellow arrows) and interacting with the crosslinkers (an example is highlighted by the green arrow). At this larger concentration, two PX molecules interact owing to the hydrophobic drug/drug interactions (see Figure 8, top left). Interestingly, the solvent-accessible surface area of the NS (see panel b) in Figure 8) is equal to 6,112 Å^2^ with the increase of 40% with respect to the initial geometry, and the radius of gyration increases by more than 90% to a value of 25.5 Å.

#### 2.2.3. NS/PX Interaction: 8 β-CDs in the NS Model and 16 PX Molecules (β-CD/Drug in a 1:2 Stoichiometry)

**Figure 9:**
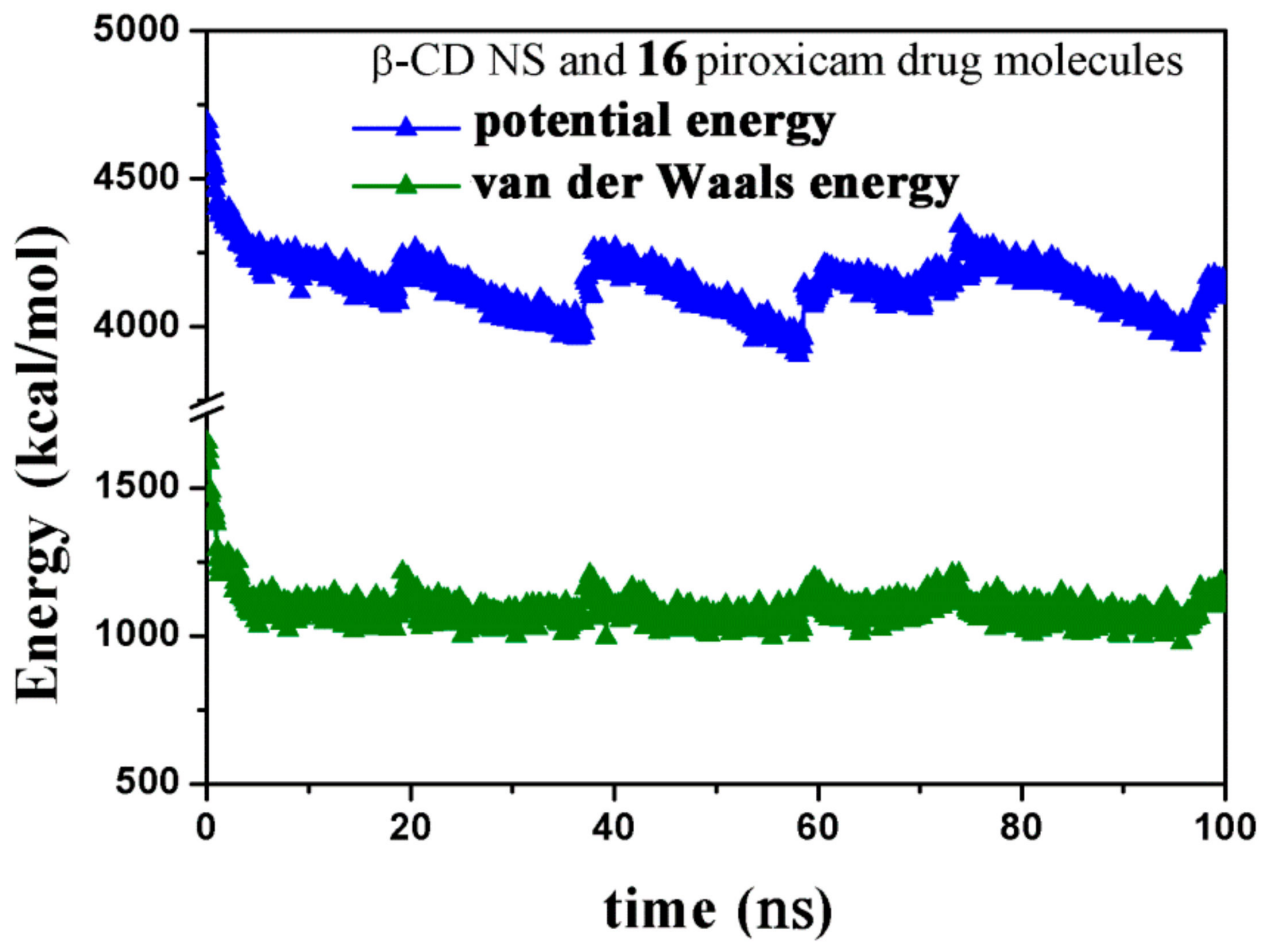
The Figure shows the potential energy and the van der Waals components calculated during the MD run for the NS interacting with 16 PX molecules.

In this Section, the results concerning the β-CD NS/drug in a 1:2 stoichiometry will be presented and discussed. After the initial energy minimization of the NS and 16 PX molecules in a random disposition, an MD simulation run was performed as before in order to understand the possible inclusion complexes or surface interactions between piroxicam and the NS. Figure 9 reports the potential energy and the van der Waals components calculated during the MD run.

**Figure 10:**
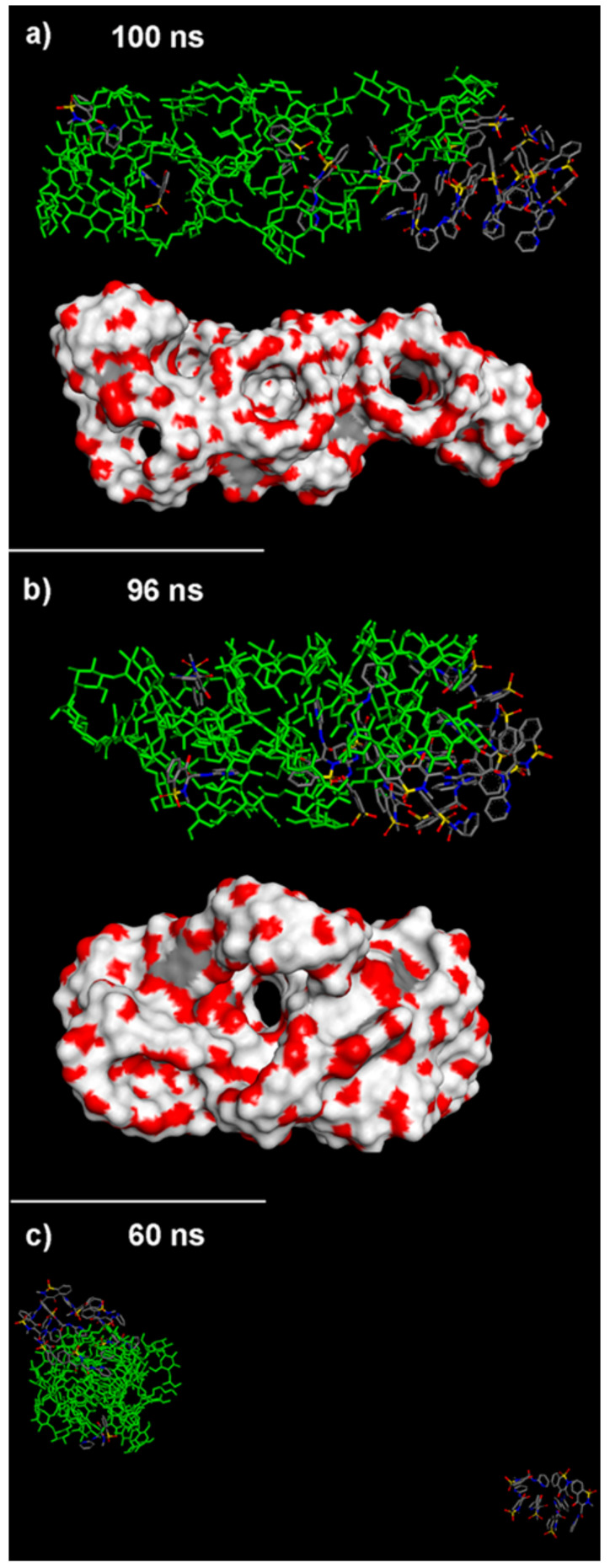
Panel (**a**) shows the optimized geometry assumed by the NS/16 PX drug molecules system at the end of the MD run lasting 100 ns and panel (**b**) displays the optimized geometry assumed by the system at 96 ns. In the top part of each panel the NS is in green for clarity and the PX molecules are colored according to the color codes of Figure 3, and in the bottom part, the solvent-accessible surface area without the PX molecules is reported for clarity (see the color codes in Figure 5). Panel (**c**) shows the geometry assumed by the system at 60 ns.

After the initial energy decrease, some conformational changes of the NS take place in order to maximize the interactions with the 16 PX molecules. The final optimized conformation assumed by the system at the end of the MD run is reported in Figure 10. During the MD run at this larger PX concentration, an equilibrium between a more compact NS conformation with a PX aggregate on its surface (see panel a) in Figure 10) and a more elongated NS conformation maximizing the interactions with the PX molecules (see panel b) in Figure 10) are observed.

The solvent-accessible surface areas of the optimized system calculated at 100 ns and 96 ns are reported in Figure 10. In particular, this area is equal to 5,664 Å^2^ with a 30% increase over the initial geometry for the conformation assumed at 100 ns, while the radius of gyration is equal to 17.0 Å, with the increase of 35%. The more compact conformation assumed by the system at 96 ns corresponds to a lower potential energy value, 2424 kcal/mol, compared to 2534 kcal/mol for the optimized geometry assumed by the system at the end of the MD run. The solvent-accessible surface area reported in Figure 10 in panel b) (bottom) without the PX molecules is equal to 5161 Å^2^, with the increase of 20%, while the radius of gyration is equal to 13.0 Å, with a 13% increase. It is interesting to note in Figure 10 the formation of a nanopore in the central part of the β-CD NS in the optimized conformation at 96 ns. It is more evident that during the MD run, the system maximizes the interaction with PX owing to high NS flexibility. In both cases, there are some inclusion complexes and a PX drug aggregate interacting on the external NS surface at 100 ns and 96 ns formed by 10 and 11 PX molecules, respectively, therefore, with about the same number of molecules that is stable in time.

Interestingly, during the MD run at 60 ns, a spherical aggregate formed by 6 molecules of the PX drug detaches from the NS surface as shown in Figure 10 (panel c)). For the animation file of the MD run, see SI. The potential energy of the system calculated during the simulation is the lowest one, indicating also an equilibrium during the MD run between the drug molecules still attached on the NS surface for the larger part of the MD run and the detached ones diffusing as a single cluster for some ns before the new adsorption on the NS surface. The solvent-accessible surface area of the system at 60 ns is equal to 5,026 Å^2^, with a 16% increase over the initial geometry, while the radius of gyration is equal to 13.0 Å, with the increase of only 4%.

#### 2.2.4. NS/PX Interaction: 8 β-CDs in the NS and 40 PX Molecules (β-CD/Drug in a 1:5 Stoichiometry)

**Figure 11:**
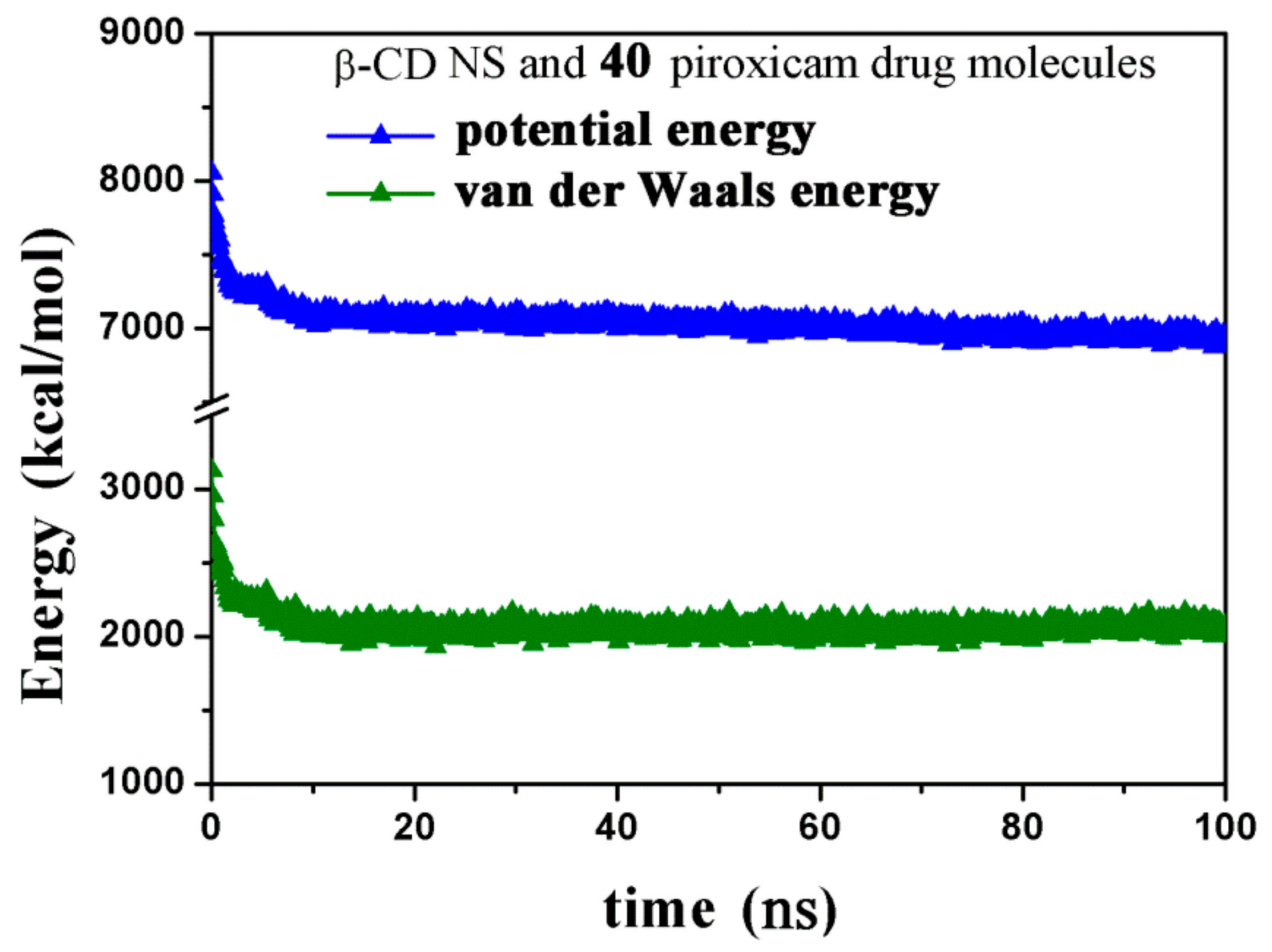
The figure shows the potential energy calculated during the MD run and the van der Waals components for the NS model and 40 PX molecules.

Finally, the results concerning the β-CD/drug in a 1:5 stoichiometry of these NS/PX systems are presented and discussed. Using the same methodology as before, the potential energy and the van der Waals components calculated during the MD run are reported in Figure 11. In this case, at the largest piroxicam concentration considered here, after the initial fast decrease of the system energy, fluctuations around average values were observed with the NS in a compact structure in contact with the PX aggregate due to drug association owing to hydrophobic π–π interactions induced by the first layer of PX molecules adsorbed on the NS surface. For the animation file of the MD run, see SI.

**Figure 12:**
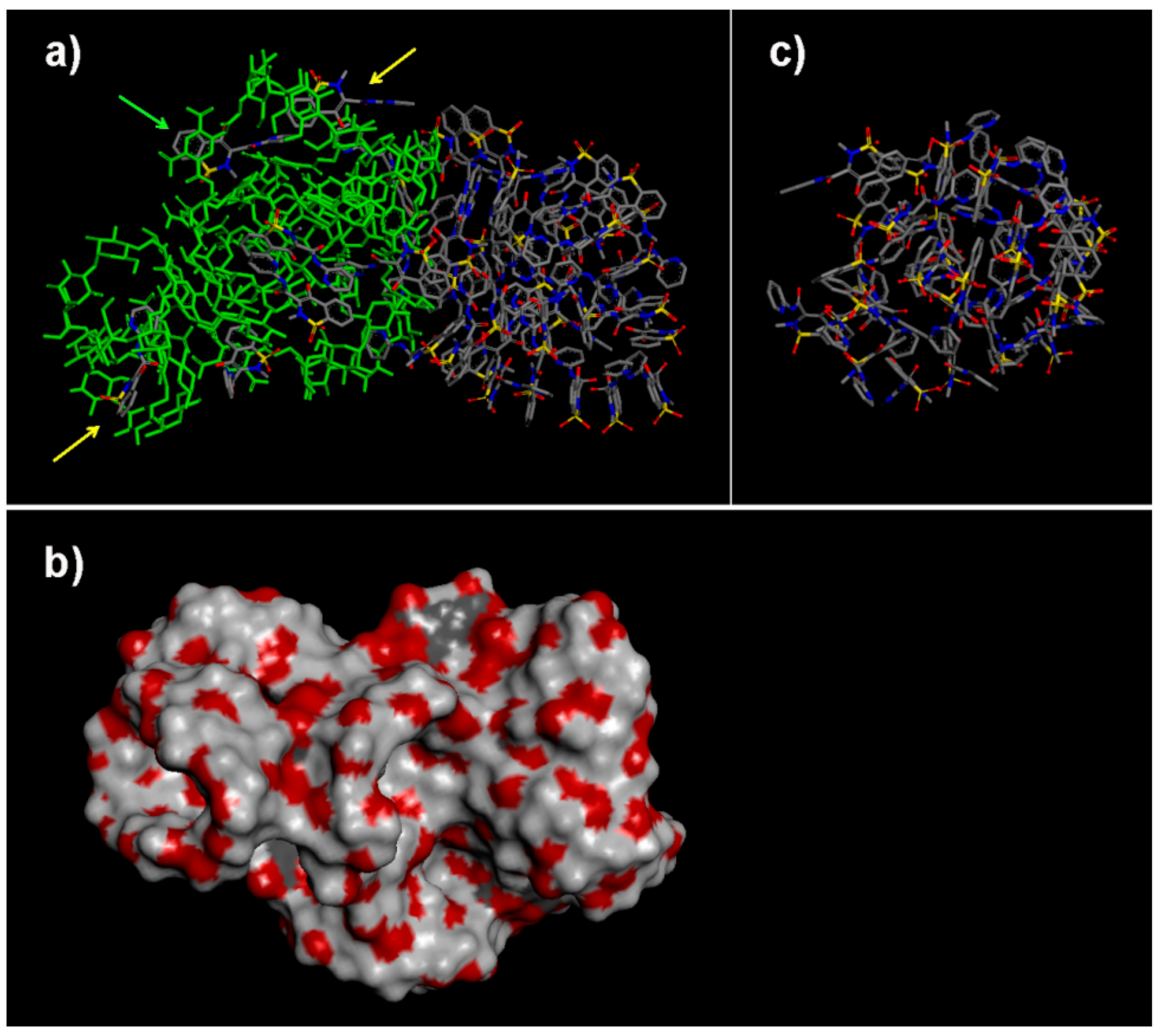
Panel (**a**) shows the optimized geometry assumed by the NS/40 PX drug molecules system at the end of the MD run displaying some host–guest inclusion complexes (see the arrows). All the other molecules are on the external surface of the NS. Panel (**b**) shows the solvent-accessible surface area without the PX molecules for clarity (see the color codes in Figure 5). Panel (**c**) shows the PX aggregate formed by 32 molecules adsorbed on the NS surface well-ordered in an almost spherical shape in detail.

The optimized conformation assumed by the system at the end of the MD run is reported in Figure 12 (panel a)).

In the optimized conformation assumed at the end of the MD run (see panel a) in Figure 12), some inclusion geometries are found, in particular, two host–guest complexes in a “type A” geometry (see the yellow arrows), the most stable one being reported in the left part of Figure 3 having the pyridyl moiety in the hydrophobic β-CD cavity, and one host–guest complex in a “type C” geometry (see the green arrow, the less stable geometry) in the right part of Figure 3 with an included phenyl. All the other drug molecules interact with the external surface of the NS. In particular, it is interesting to note the almost spherical aggregate that is nucleated on the external surface after the initial favorable interaction of the first layer of adsorbed PX molecules. β-CD acts as a surface of the nucleation process which subsequently takes place thanks to π–π interactions between the drug molecules. Considering this final optimized geometry, a self-ordered structure is clearly seen in the right part of panel a) of Figure 12, with a parallel arrangement of the aromatic rings of the drug molecules producing an almost spherical aggregate formed by 32 PX molecules represented in another orientation in panel c). The conformation of the β-CD NS having the PX aggregate adsorbed is compact and, in fact, its radius of gyration is equal to 13.5 Å, larger only by 7% with respect to the isolated NS, and the solvent-accessible surface area is equal to 4,920 Å^2^ (see panel b) in Figure 12), larger by 14% than in the isolated NS. Interestingly, in this case, the calculated increase was smaller than in the simulations at relatively smaller PX concentrations.

## 3. Materials and Methods

The interaction of PX with the β-CD NS model was studied using molecular mechanics (MM) and molecular dynamics (MD) methods at the constant temperature of 300 K. The MM and MD simulations are performed using the consistent valence force field (CVFF) [33], and the Materials Studio and Insight II/Discover packages [34] adopting the same simulation protocol as proposed in yjr previous work [20,21,22,23]: after the initial energy minimization, MD runs were carried out until the equilibrium state was achieved followed by final geometry optimizations of the final configuration and of some conformations assumed during the MD run. The PX structure (see Scheme 1) was generated using the Insight II/Discover Module Builder and the final most stable geometry (Figure 3) was obtained after an MD run lasting for 1 ns, with the final optimization of different conformations assumed by the system during the MD run. At first, the possible β-CD/PX inclusion compounds in a 1:1 and in a 2:1 stoichiometry were studied. Then, the interaction between the β-CD NS model and PX was considered. The structure of the β-CD NS model reported by Raffaini [30] was generated by linking 8 β-CD (Model 2 in [30]) with pyromellitic dianhydride (PMA): each β-CD carries two PMA linking agents bound to a primary hydroxyl at diametrically opposite sides of the macrocycle, linking also two topologically distant β-CDs. The drug molecules were initially placed in a random distribution cell far from to the NS surface in order to study the interactions without assuming any a priori inclusion complexes. The interaction was studied considering 4, 8, 16, and 40 PX molecules randomly distributed around the NS model in a 220 Å cubic cell with a varying CDs/PX ratio. All simulations were performed in implicit water using a distance-dependent dielectric constant with periodic boundary conditions. All energy minimizations were carried out up to an energy gradient < 4 × 10^−3^ kJ mol^−1^ Å^−1^. The MD simulations were performed in an NVT ensemble (canonical ensemble, see Abbreviations) at a constant temperature (300 K) controlled using a Berendsen thermostat. The integration of dynamical equations was carried out with the Verlet algorithm using a time step of 1 fs and the instantaneous coordinates were periodically saved for further analysis. The MD runs in implicit water lasted for 10 ns in Section 2.1 and 100 ns for the NS systems in Section 2.2. During the MD runs, the time evolution of the potential energy and of the van der Waals components was calculated in order to monitor the significant conformational changes.

## 4. Conclusions

Drug concentration plays an important role in the interaction with drug carriers affecting the kinetics of the release process and the toxicology effects. Hydrophobic drugs can be solubilized in water through CD in order to enhance their bioavailability. Solubility of PX, an important nonsteroidal anti-inflammatory drug, is enhanced by β-CDs. In the present work, MD simulation of their host–guest complexes in a 1:1 and a 2:1 stoichiometry are reported, with results in good agreement with the NMR experimental literature data. Different host–guest inclusion geometries were obtained considering both native β-CD and crosslinked β-CD nanosponges (NS). When PX interacts with an NS, at smaller concentration, the drug molecules form inclusion complexes and feature some interactions on the external NS surface, dynamically forming some H-bonds or hydrophobic interaction with the PMA crosslinker, while at larger concentrations, they also aggregate on the NS surface, forming spherical droplets. Therefore, the β-CD NS enhances the solubility of hydrophobic PX. At small drug concentration, during the MD runs, the NS shows equilibrium between an extended structure, when the NS maximizes the interactions with drug molecules exposing a larger surface area, and a more compact structure, also forming a temporary nanopore when drug/drug self-aggregation due to π–π interactions takes place on its external surface. The noncovalent interactions are favored by the great NS flexibility, enhancing solubilization and PX transport. The drug concentration plays an important role. At larger concentrations, a spherical PX nanoaggregate is observed on the external NS surface besides drug inclusion complexes, because the first layer of adsorbed PX molecules induces aggregation of other hydrophobic molecules, the NS acting as a nucleation surface. During an MD run, an equilibrium is also observed between the drugs adhered on the NS surface and the detached ones in a small aggregate that diffuses before subsequent adsorption on the NS surface. Finally, it is important to highlight that the β-CD/drug and the drug/drug interactions and association will likely also affect the kinetics of the release process, depending on the site of interaction and on the flexibility of the crosslinked β-CDs. It will be important to theoretically investigate this system at a specific concentration to model the release process in order to better understand the specific interactions and the geometry and stability of the aggregates, which may affect the kinetics of the release process. Theoretical and experimental studies of the β-CD NS/PX interaction in a specific stoichiometry and drug release are in progress and will be published in a separate paper.

## Figures and Tables

**Table 1:** Potential energy and interaction energy in kJ/mol calculated in the optimized geometries assumed at the end of the MD runs for the most and the less stable final geometries. Intermolecular β-CD/PX H-bonds and intramolecular β-CD H-bonds are reported.

Geometry	*E_pot_* (kJ/mol)	*E_int_* (kJ/mol)	Intermolecular H-Bonds	Intramolecular β-CD H-Bonds
A) Most stable	766.5	−173.2	1 bidentate (with II rim)	16
B) Metastable	769.4	−170.3	1 (with I rim)	17
C) Less stable	776.6	−163.1	1 (with II rim)	8
D) Metastable	771.5	−168.1	1 bidentate (with II rim)	12

**Table 2:** Interaction energy of the optimized geometries assumed at the end of the MD runs for the most stable and the less stable final geometries in a 2:1 stoichiometry. Intermolecular β-CD/PX and β-CD H-bonds and intramolecular β-CD H-bonds are also reported.

Geometry	*E_pot_* (kJ/mol)	*E_int_* (kJ/mol)	Intermolecular β-CD/PX H-bonds	Intermolecular β-CD H-bonds	Intramol.first β-CD H-bonds	Intramol.second β-CD H-bonds
A) Most stable	233.4	−381.7	1 (with II rim)	5	9	8
B) Less stable	247.6	−322.8	2 (with II rim)	4	18	15
C) Metastable	241.4	−348.8	1 (with II rim)	3	13	15
D) Metastable	242.8	−342.5	2 (1 with I and II rims)	5	16	18

## Supplementary Materials

Supplementary data can be found in the online version.

## Abbreviations

MM	molecular mechanics
MD	molecular dynamics
β-CD	β-cyclodextrin
PX	piroxicam
PMA	pyromellitic dianhydride
NS	nanosponge
β-CD NS	β-cyclodextrin nanosponge
NVT ensemble	Number of particles, Volume and Temperature are constant
