# Supplementary material for: Understanding Surface Interaction and Inclusion Complexes between Piroxicam and Native or Crosslinked β-Cyclodextrins: The Role of Drug Concentration
Source: Molecules. 2020 Jun 19;25(12):2848. doi: 10.3390/molecules25122848 (PMC7355541; doi:10.3390/molecules25122848)
Supplement: Supplementary file 1 [file molecules-25-02848-s001.pdf]

Understanding surface interaction and inclusion complexes between piroxicam and native or crosslinked β-Cyclodextrins: the role of the drug concentration

Giuseppina Raffaini [1,2]*, Fabio Ganazzoli [1,2]

[1] Department of Chemistry, Materials, and Chemical Engineering "Giulio Natta", Politecnico di Milano, Piazza L. Da Vinci 32, 20131 Milano (Italy); fabio.ganazzoli@polimi.it (F.G.)
[2] INSTM, National Consortium of Materials Science and Technology, Local Unit Politecnico di Milano, Milano – Italy
* Correspondence: giuseppina.raffaini@polimi.it ; Tel.: +39-02-23993068 (G.R.)

† *Dedicated to the memory of the late Professor Giuseppe Allegra*

Table of Contents

2.1.1. β-CD/PX Interaction: Complex Formation in a 1:1 Host-Guest Stoichiometry

Figure S1. At left side view and at right top view with H-bonds reported for the 1:1 *host-guest* complexes formed by PX and β-CDs, in the metastable optimized geometries found after the MD runs indicated respectively in Table 1 as geometry B (above) and geometry D (below). The β-CD is in green. The piroxicam molecule is colored by atoms. Color code: C atoms are grey, and O atoms are red, N atoms in blue, S atoms in yellow, hydrogens in white. At left the H atoms are omitted for clarity. At right the H-bonds are indicated by dotted light blue line…………………………………………………...S3

2.1.2 β-CD/PX Interaction: Complex Formation in a 2:1 Host-Guest Stoichiometry

Figure S2. Side view of the initial *non-optimized* geometries considering the most (above) and the less stable (below) β-CD/PX complex previously studied in *Section 2.1.2*, facing it with a second β-CD with the primary (at left, A) and C) and the secondary rim (at right, B) and D), as indicated in the two panels with the secondary rim and at right near the primary rim of the second β-CD considered in order to study possible complexes in a 2:1 stoichiometry……………………………………………………………..S4

Figure S3. Side view of the final metastable *optimized* geometries in a 2:1 stoichiometry found after the MD runs considering β-CD/PX complexes indicated in Table 2 in *Section 2.1.2.*; in particular for the geometry C see panel a), for the geometry D see panel b). The β-CD of the first 1:1 complex is in green, the second one facing the secondary rim is in light blue. The piroxicam molecule is colored by atoms. Color code: C atoms are grey, and O atoms are red, N atoms in blue, S atoms in yellow, hydrogens in white. At left the H atoms are omitted for clarity……………………………………………………………S5

We can follow the MD run lasting 10 ns starting from the Type of Geometry A) and C) in Figure S1 in the following animation file:

MD run_ Type A host-gest dimer_dimer_SS_10ns.avi,

MD run_ Type C host-gest dimer_dimer_SS_10ns.avi, respectively.

2.2.1 Interaction NS/PX: 8 β-CDs in NS model and 4 PX molecules [β-CDs/drug in a 2:1 stoichiometry]

We can follow the MD run lasting 100 ns in the following animiation file:

MD run_ BetaCD PMA NS_4 Piroxicam drugs_100ns.avi.

2.2.3. Interaction NS/PX: 8 β-CDs in NS model and 16 PX molecules [β-CDs/drug in a 1:2 stoichiometry]

We can follow the MD run lasting 100 ns in the following animiation file:

MD run_ BetaCD PMA NS_16 Piroxicam drugs_100ns.avi

2.2.4 Interaction NS/PX: 8 β-CDs in NS model and 40 PX molecules [β-CDs/drug in a 1:5 stoichiometry]

We can follow the MD run lasting 100 ns in the following animiation file:

MD run_ BetaCD PMA NS_40 Piroxicam drugs_100ns.avi.

2.1.1. β-CD/PX Interaction: Complex Formation in a 1:1 Host-Guest Stoichiometry

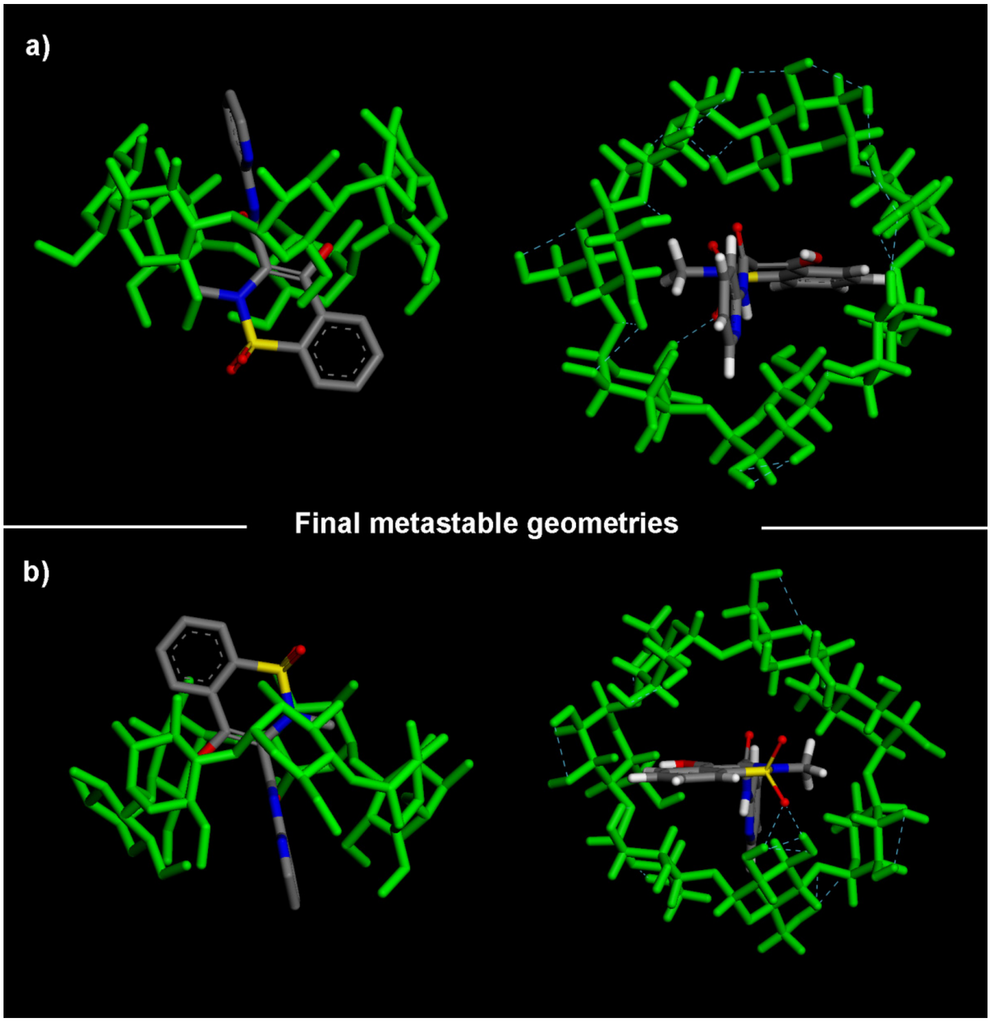

Figure S1. At left side view and at right top view with H-bonds reported for the 1:1 *host-guest* complexes formed by PX and β-CDs, in the metastable optimized geometries found after the MD runs indicated respectively in Table 1 as geometry B (above) and geometry D (below). The β-CD is in green. The piroxicam molecule is colored by atoms. Color code: C atoms are grey, and O atoms are red, N atoms in blue, S atoms in yellow, hydrogens in white. At left the H atoms are omitted for clarity. At right the H-bonds are indicated by dotted light blue line.

2.1.2 β-CD/PX Interaction: Complex Formation in a 2:1 Host-Guest Stoichiometry

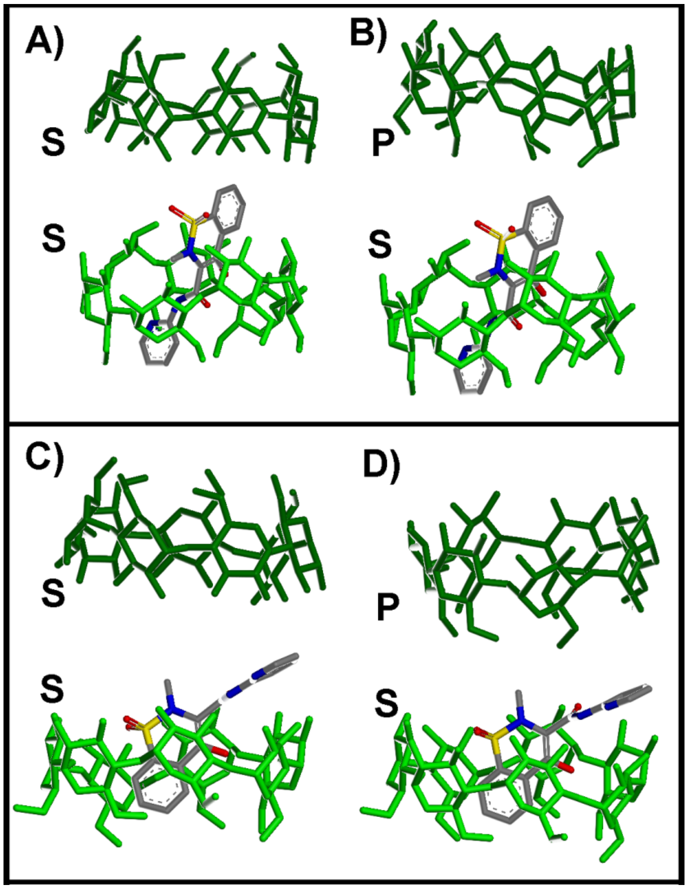

Figure S2. Side view of the initial *non-optimized* geometries considering the most (above) and the less stable (below) β-CD/PX complex previously studied in *Section 2.1.2*, facing it with a second β-CD with the primary (at left, A) and C) and the secondary rim (at right, B) and D), as indicated in the two panels with the secondary rim and at right near the primary rim of the second β-CD considered in order to study possible complexes in a 2:1 stoichiometry. S2

Color code: the carbon atoms of the first β-CD in the host-guest complex is in green, the second one in dark green. Piroxicam atoms are colored by atoms: carbon in grey, oxygen in red; sulfur in yellow, nitrogen blue. Hydrogen atoms are omitted for clarity.

We can follow the MD run lasting 10 ns starting from the Type of Geometry A) and C) in Figure S1 in the following animation files:

MD run_ Type A host-gest dimer_dimer_SS_10ns.avi,

MD run_ Type C host-gest dimer_dimer_SS_10ns.avi, respectively.

2.1.2 β-CD/PX Interaction: Complex Formation in a 2:1 Host-Guest Stoichiometry

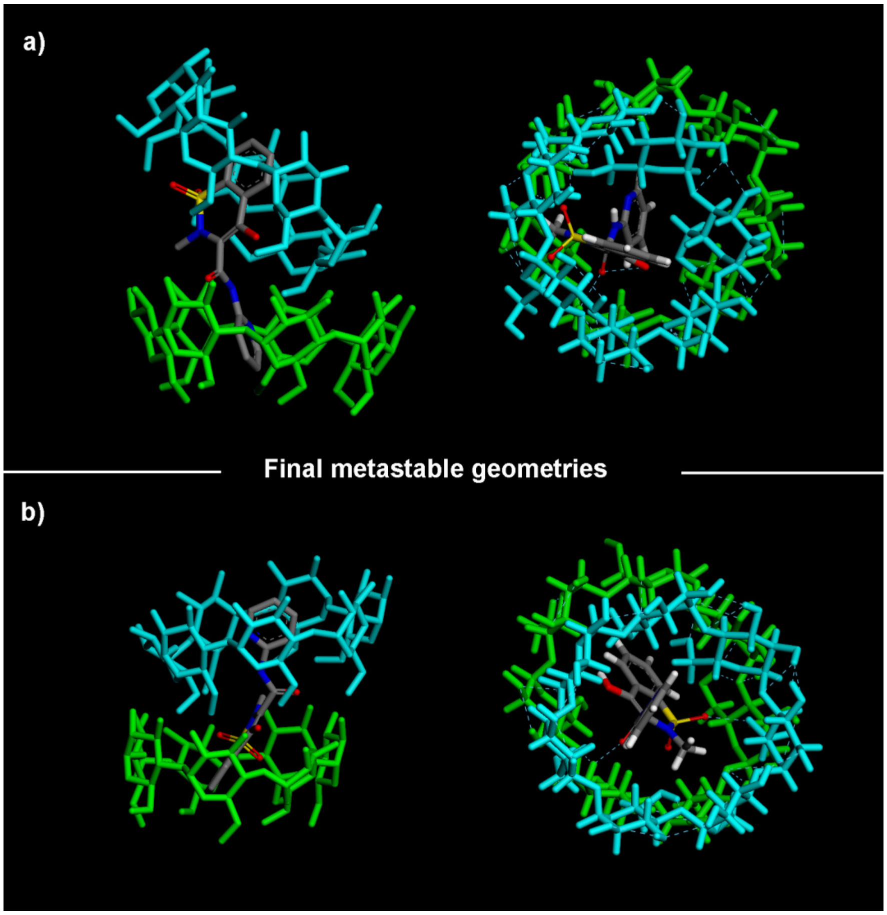

Figure S3. Side view of the final metastable *optimized* geometries in a 2:1 stoichiometry found after the MD runs considering β-CD/PX complexes indicated in Table 2 in *Section 2.1.2.*, in particular for the geometry C see panel a), for the geometry D see panel b). The β-CD of the first 1:1 complex is in green, the second one facing the secondary rim is in light blue. The piroxicam molecule is colored by atoms. Color code: C atoms are grey, and O atoms are red, N atoms in blue, S atoms in yellow. At left the H atoms are omitted for clarity.
